# Novel Amplicon-Based Sequencing Approach to West Nile Virus

**DOI:** 10.3390/v15061261

**Published:** 2023-05-27

**Authors:** Moussa Moïse Diagne, Marie Henriette Dior Ndione, Giulia Mencattelli, Amadou Diallo, El hadji Ndiaye, Marco Di Domenico, Diawo Diallo, Mouhamed Kane, Valentina Curini, Ndeye Marieme Top, Maurilia Marcacci, Maïmouna Mbanne, Massimo Ancora, Barbara Secondini, Valeria Di Lollo, Liana Teodori, Alessandra Leone, Ilaria Puglia, Alioune Gaye, Amadou Alpha Sall, Cheikh Loucoubar, Roberto Rosà, Mawlouth Diallo, Federica Monaco, Ousmane Faye, Cesare Cammà, Annapaola Rizzoli, Giovanni Savini, Oumar Faye

**Affiliations:** 1 Virology Department, Institut Pasteur de Dakar, Dakar BP220, Senegal; marie.ndione@pasteur.sn (M.H.D.N.); mouhamedkane2015@gmail.com (M.K.); maimounambanne16@gmail.com (M.M.); amadou.sall@pasteur.sn (A.A.S.); ousmane.faye@pasteur.sn (O.F.); oumar.faye@pasteur.sn (O.F.); 2 Istituto Zooprofilattico Sperimentale dell’Abruzzo e del Molise, 64100 Teramo, Italy; giulia.mencattelli@unitn.it (G.M.); m.didomenico@izs.it (M.D.D.); v.curini@izs.it (V.C.); m.marcacci@izs.it (M.M.); m.ancora@izs.it (M.A.); b.secondini@izs.it (B.S.); v.dilollo@izs.it (V.D.L.); l.teodori@izs.it (L.T.); a.leone@izs.it (A.L.); i.puglia@izs.it (I.P.); f.monaco@izs.it (F.M.); c.camma@izs.it (C.C.); g.savini@izs.it (G.S.); 3 Centre Agriculture Food Environment, University of Trento, 38010 San Michele all’Adige, Italy; roberto.rosa@unitn.it; 4 Research and Innovation Centre, Fondazione Edmund Mach, 38010 San Michele all’Adige, Italy; annapaola.rizzoli@fmach.it; 5 Epidemiology, Clinical Research and Data Science Department, Institut Pasteur de Dakar, Dakar BP220, Senegal; amadou.diallo@pasteur.sn (A.D.); ndeyemarieme.top@pasteur.sn (N.M.T.); cheikh.loucoubar@pasteur.sn (C.L.); 6 Medical Zoology Department, Institut Pasteur de Dakar, Dakar BP220, Senegal; elhadji.ndiaye@pasteur.sn (E.h.N.); diawo.diallo@pasteur.sn (D.D.); alioune.gaye@pasteur.sn (A.G.); mawlouth.diallo@pasteur.sn (M.D.)

**Keywords:** West Nile virus, lineages, next-generation sequencing, amplicon-based sequencing

## Abstract

West Nile virus is a re-emerging arbovirus whose impact on public health is increasingly important as more and more epidemics and epizootics occur, particularly in America and Europe, with evidence of active circulation in Africa. Because birds constitute the main reservoirs, migratory movements allow the diffusion of various lineages in the world. It is therefore crucial to properly control the dispersion of these lineages, especially because some have a greater health impact on public health than others. This work describes the development and validation of a novel whole-genome amplicon-based sequencing approach to West Nile virus. This study was carried out on different strains from lineage 1 and 2 from Senegal and Italy. The presented protocol/approach showed good coverage using samples derived from several vertebrate hosts and may be valuable for West Nile genomic surveillance.

## 1. Introduction

The threat from new re-emerging viruses has markedly increased in recent decades due to population growth, urbanization, and the expansion of global travel, facilitating the rapid spread of infection during an outbreak. West Nile virus (WNV), an arbovirus belonging to the flavivirus genus, was firstly isolated in 1937 in Uganda [1] before spreading throughout the world [2]. The enzootic cycle includes mosquitoes and several vertebrate species including birds, allowing long-distance viral spread during migratory seasons [3,4]. Humans are considered WNV dead-end hosts because no human-to-mosquito transmission has been reported yet [5]. Most WNV infections are asymptomatic or may develop into self-limited febrile illness, but a very small percentage of cases progress to neuroinvasive disease with a range of symptoms and occasionally death [6,7].

Before 1990, WNV disease was considered to have a minor public health impact with only sporadic human cases. Since the first outbreaks reported in Algeria and Romania in 1994 and 1996, the virus has diffused to cause large epidemics in North America, Northern African, and Western and Eastern European countries [7].

In Italy, areas with either proven active asymptomatic WNV circulation or high probability of human infection have been previously reported [8,9], and an increasing number of neuroinvasive human infections have been described [10,11].

In Africa, little evidence of WNV epidemics has been noted. In Senegal, where WNV was first isolated in an acute human case in 1970, the virus has also been detected in mosquitoes, birds, horses, and human samples. From 2012 to 2021, active WNV circulation in mosquitoes and humans was documented following a reintroduction event from Europe [12].

WNV exhibits great genetic diversity with currently eight different lineages (excluding Koutango virus) circulating in the world [13]. Lineages 1 (WNV-L1) and 2 (WNV-L2) are the ones causing the main public health concern [7,12]. Genetic characterization of the strains detected yield potential tracking of the routes of the introduction of viruses, which is a particular interest for public health authorities in designing surveillance and countermeasures plans.

Genome sequencing of viruses has proven to be critical in the management of epidemics. Many approaches can be used to obtain viral whole genomes: (i) propagation with cell cultures followed by nucleic acids metagenomic (mNGS); (ii) hybrid capture using specific biotinylated probes; and (iii) a multiplex PCR-based target enrichment or amplicon-based protocol. This last approach became the most used one for the SARS-CoV-2 genomic surveillance during the COVID-19 pandemic due to its applicability in a wide range of input titers, yielding directly sequenced clinical samples, as well as its high specificity and scalability under resource-limited conditions with lower costs [14,15,16,17].

Due to the wide range of WNV hosts, many One Health studies focus on WNV. As genomic data are key information for understanding the mechanisms of the emergence and circulation of this virus, it is crucial to develop a rapid, reliable, and cost-effective sequencing tool that is more accessible than isolation methods or mNGS.

We describe here the development and evaluation of a whole-genome amplicon-based sequencing approach for WNV-L1 and WNV-L2 using Illumina technology in different types of vertebrates and mammals from Senegal and Italy.

## 2. Materials and Methods

### 2.1. Primers Design for Tiled Amplicon-Based Sequencing Systems for West Nile Virus

Primer design was made in IPD using a web-based tool entitled Primal Scheme [18] in order to obtain two non-overlapping pools of WNV targeting primers to perform multiplexed PCR reactions, generating approximately 400 bp amplicons tiled along the targeted genome. A WNV reference genome (accession number: NC009942) was chosen as the template. An alignment of WNV whole-genome sequences available on Genbank representative of all WNV lineages in both Africa and Europe was then used to identify nucleotide mismatches for potential correction at ambiguous sites of each primer to ensure both good coverage and high specificity for diverse WNV lineages. Overall, the approach used was a two-pool multiplex amplicon-based sequencing.

### 2.2. West Nile Virus Primer Pools Validation

Validation of the primer sets followed several steps: (i) inclusivity test by sequencing attempts on several WNV-L1 and WNV-L2 strains; (ii) specificity and sensitivity tests by sequencing attempts on several flaviviruses and other arboviruses, as well as serial dilutions of WNV-L1 and WNV-L2 culture isolates; and (iii) final validation by sequencing confirmed positive WNV samples derived from different species of vertebrates and mosquitoes from Italy and Senegal.

#### 2.2.1. Sequencing of WNV-L1 and WNV-L2 Isolates

The designed primer systems were challenged for amplicon-based whole-genome sequencing of well-characterized WNV-L1 and WNV-L2 isolates from Senegal and Italy. The experiments were undertaken by both the teams in Senegal and Italy with their local isolates. WNV-L1 (n = 10) and WNV-L2 (n = 8) well-characterized viral isolates from both countries were used to assess the ability of the designed primer pools for whole-genome amplicon-based sequencing. WNV strains from Senegal were obtained after infection of C6/36 monolayer cells with homogenized mosquito pools as previously described [12]. Isolates from Italy were obtained from birds’ internal organ homogenates after two to three passages on Vero monolayer cell lines, followed by an infection on C6/36 cell lines. A genome coverage of 95% and above was targeted.

#### 2.2.2. Specificity and Sensitivity of the WNV Amplicon-Based Sequencing Systems

The second step was to assess specificity by performing the experiment on several other arboviruses: Rift Valley fever virus (RVFV); yellow fever virus (YFV); Zika virus (ZIKV); dengue 2 virus (DENV-2); Wesselsbron virus (WSLV); Kedougou virus (KDGV); Usutu virus (USUV); and chikungunya virus (CHIKV). The sensitivity of the approach was evaluated using serial dilutions of WNV-L1 and WNV-L2 culture isolates at different concentrations (10^6^–10^2^ RNA copy/μL). Each concentration was sequenced in triplicate.

#### 2.2.3. Validation on Confirmed Positive WNV Samples

Finally, sequencing attempts on both WNV-L1 and WNV-L2 positive samples from mosquitoes, birds, and horses from Italy and Senegal were conducted. The CT values of the samples were confirmed by RT-qPCR using a consensus WNV assay [6] in Senegal and a molecular WNV sub-typing assay [19] in Italy, prior to proceeding to the sequencing.

#### 2.2.4. Next-Generation Sequencing and Genome Assembly

Viral RNAs were extracted using the QIAamp viral RNA mini-kit (QIAGEN, Hilden, Germany) and were reverse-transcribed into cDNAs using the Superscript IV Reverse Transcriptase enzyme (ThermoFisher Scientific, Waltham, MA, USA). The synthesized cDNAs served as templates for direct amplification to generate approximately 400 bp amplicons tiled along the genome using two non-overlapping pools of WNV targeting primers at 10 nM and Q5^®^ High-Fidelity 2X Master Mix (New England Biolabs) with the following thermal cycling protocol: 98 °C for 30 s; 35 cycles of 95 °C for 15 s and 65 °C for 5 min; and a final cooling step at 4 °C.

In Senegal, libraries were then synthesized by tagmentation using the Illumina DNA Prep kit and the IDT^®^ for Illumina PCR Unique Dual Indexes. After a cleaning step with the Agencourt AMPure XP beads (Beckman Coulter, Indianapolis, IN), libraries were quantified using a Qubit 3.0 fluorometer (Invitrogen Inc., Waltham, MA, USA) for manual normalization before pooling in the sequencer. Cluster generation and sequencing were conducted with ab Illumina MiSeq instrument with 2 × 300 nt read length. Consensus genomes were generated using the nextflow-based nf-core viral reconstruction pipeline (https://github.com/nf-core/viralrecon, accessed on 20 January 2023) from the standardized nf-core pipelines [20,21]. The versions of nextflow and viralrecon used were v21.10.6 and v2.5, respectively. In Italy, amplified DNA was diluted to obtain a concentration of 100–500 ng, then used for library preparation with an Illumina DNA prep kit, and sequenced with a NextSeq 500 (Illumina Inc., San Diego, CA, USA) using a NextSeq 500/550 Mid Output Reagent Cartridge v2 for 300 cycles with standard 150 bp paired-end reads. After quality control and trimming with the Trimmomatic v0.36 (Usadellab, Düsseldorf, Germany) [22] and FastQC tool v0.11.5 (Bioinformatics Group, Babraham Institute, Cambridge, UK) [23,24], reads were de novo assembled using SPADES v3.11.1 (Algorithmic Biology Lab, St Petersburg, Russia) [25]. The contigs obtained were analyzed with BLASTn to identify the best match reference. Mapping of the trimmed reads was then performed using the iVar computational tool [26] to obtain a consensus sequence. 

## 3. Results

### 3.1. West Nile Virus Oligonucleotide Primers Sets

A first multiplex primer system was designed based on a WNV-L1 reference genome (accession number: NC009942), generating a set of 35 oligonucleotide primer pairs that amplify overlapping products spanning almost the whole WNV genome.

The primers set (set A) was subsequently compared to an alignment of 15 sequences representing the different WNV lineages (Table S1). Degeneration was then added in relevant ambiguous sites on each primer in order to cover a maximum of lineages while trying to maintain a balance for specificity. The list of WNV primers in set A can be found in Table 1. We should notice that two extra primers (KOUV_2_RIGHT and KOUV_7_LEFT) were incorporated into set A to potentially extend the sequencing to Koutango virus, even if this work was not carried out in this study.

A second primer set (set B) was designed based on a WNV-L2 reference genome (accession number: MH021189) and was compared with an alignment of 82 WNV-L2 sequences (Table S2) to capture the diversity within the lineage. The list of WNV primers in set B can be found in Table 1.

### 3.2. WNV Primers Sets Validation

#### 3.2.1. Validation of Set A

##### Inclusivity Test

After the design of set A, seven WNV-L1 and three WNV-L2 isolates from Senegal were selected, and three viral culture supernatants for each lineage from three different Italian regions were processed for amplicon-based sequencing in triplicate. Overall, tiled amplicon whole-genome sequencing undertaken on both strains from Senegal and Italy yielded 99–100% horizontal coverage with genome length between 10,961 nt and 11,018 nt for WNV-L1 and between 10,914 nt and 10,926 nt for WNV-L2 (Table 2).

##### Sensitivity Test

One representative isolate of each lineage, i.e., WNV 15217 (accession number: FJ483548) and WNV Thessaloniki_MC82m/2018 (accession number: MN652880) for WNV-L1 and WNV-L2, respectively, was selected to evaluate the detection limit of the set A primers under optimal conditions. Serial dilutions from 10^6^ to 10^2^ cp/µL were processed in triplicate for sequencing. The set A primers were able to detect more than 95% of the total WNV-L1 genome up to 10^4^ cp/µL. At 10^3^ cp/µL, the horizontal coverage was between 91% and 94%, while at 10^2^ cp/µL, 80 to 82% of the WNV-L1 sequence was completed. However, poor coverage was observed in the WNV-L2 samples (between 17% and 35% completeness) as shown in Table 3.

##### Specificity Test

Amplicon-based whole-genome sequencing with set A primers was conducted on six flavivirus species (YFV, ZIKV, DENV-2, WSLV, KDGV, USUV), as well as RVFV and CHIKV, in order to assess the specificity of this WNV targeted approach. All the samples failed the bowtie2 1000 mapped-read threshold and no consensus genome could be assembled.

##### WNV Set A Primers Validation on Real Homogenates

Thirty-one (31) WNV-L1 and fifty-four (54) WNV-L2 homogenates with known Ct values by RT-qPCR were selected for targeted sequencing using the set A primers. Homogenates were obtained from mosquito pools and the internal organs of birds with low to high viral loads.

Among WNV-L1 homogenates, horizontal coverage was between 34% and 100%. A total of 35% of the samples reached above 95% horizontal coverage and about 65% of samples for 90% horizontal coverage. Most complete genomes had Ct values between 16 and 28. However, we also noted that among the least well-covered samples, Ct values ranged from 25 to 35, highlighting that factors other than the viral load could be involved. Additionally, five samples were WNV-L1/WNV-L2 co-infections, and the amplicon-based approach yielded from 87% to 96% WNV-L1 horizontal coverage, even when WNV-L2 had a higher viral load. Relatively correct coverage (between 74% and 92%) was obtained from other four samples from mosquitoes trapped in Senegal, for which viral co-infections with either alphaviruses, mesoniviruses, or flaviviruses were reported. All these results are summarized in Table 4.

Regarding WNV-L2 homogenates, experiments undertaken with the set A primers were consistent with the data from inclusivity and specificity tests. Indeed, less than 6% of the samples processed had above 95% of the genome covered (3 out 54), and 87% had ≤64% horizontal coverage, regardless of the viral load (Table 5).

#### 3.2.2. Validation of Set B

##### Inclusivity Test

Five WNV-L2 isolates from Italy were selected to assess the set B primers. A total of 100% horizontal coverage was obtained for all the strains after sequencing on an Illumina MiSeq (Table 6).

##### Sensitivity Test

In order to identify the set B primers’ detection limit under optimal conditions, serial dilutions from 10^6^ to10^2^ cp/μL of the strain WNV Thessaloniki_MC82m/2018 (accession number: MN652880) were processed in triplicate for sequencing (except the to10^2^ cp/μL concentration, which was carried out in duplicate due to insufficient volume during the experiment). A total of 100% horizontal coverage was obtained between 10^6^ to10^3^ cp/μL, while the two replicates for to10^2^ cp/μL covered 93% and 95% of the genome, as shown by Table 7.

##### Specificity Test

Similar to the test conducted for set A, no amplification was observed using set B on the six flavivirus species mentioned above, as well as RVFV and CHIKV.

##### WNV Set B Primers Validation on Real Homogenates

Fifteen WNV-L2 homogenates from Italy with known CT values by RT-qPCR were selected for targeted sequencing using the set B primers. Homogenates were obtained from mosquito pools, as well as the internal organs of birds and horses with low to high viral loads. Overall, horizontal coverage between 97% and 100% was obtained on 14 out of 15 homogenates (93.3% with horizontal coverage > 95%). Only the horse sample exhibited 93% horizontal coverage. This sample was also the one with the lowest viral load (CT value: 35). All these results are summarized in Table 8.

#### 3.2.3. Validation of Set A + B

In order to obtain a system able to efficiently sequence both WNV-L1 and WNV-L2 strains, the first set of primers (set A) was combined with the second one (set B) in equal volume. The new system, set A + B primers, was evaluated and compared in parallel with set A and set B after sequencing the WNV-L1 (n = 4) and WNV-L2 (n = 7) positive samples from internal organs of birds and horses, as well as mosquito homogenates, at different CT values (Table 9).

In WNV-L1 samples, no loss of sensitivity was observed between set A and set A + B for all the samples tested. Notably, for one sample from a yellow-legged gull at CT value 25, a gain of sensitivity was observed at 88% horizontal coverage using set A to 93% using set A + B joined. In the same way, sequencing conducted on WNV-L2 samples worked just as well with set B as with set A + B, regardless of Ct values. Indeed, almost 72% of the samples had 100% full genome (n = 5 out of 7).

## 4. Discussion

NGS is now an essential tool in the study of infectious diseases, both at the fundamental level and in its application to public health. The COVID-19 pandemic has thus been a patent example of the importance of being able to obtain information on the genetic signature of pathogens in real time. However, it should be noted that sequencing technology, and in particular whole-genome sequencing, remains an expensive approach with significant experimental constraints (for instance, the host genome background with a relatively lower amount of genetic material of the pathogen of interest in clinical specimens) in order to have some quality of data generated. A multiplex PCR-based target enrichment or amplicon-based protocol [14] was mostly used to overcome these challenges during SARS-CoV-2 genomic surveillance, yielding more than 14 million genomes in the GISAID platform at the time of writing this manuscript [27].

WNV is becoming a major health problem in Europe and cases have also recently been detected in Africa [7,12].

WNV cases are mainly due to lineages 1 and 2. The mechanisms of diffusion of viral strains, in particular by the migratory movements of birds, are actively studied. The genetic characterization of the identified strains allow better control of the dissemination routes for effective sanitary measures. NGS showed the persistence of a WNV strain after winter in Andalusia in Spain, suggesting endemicity with potential future epidemics in the area [28]. Another recent genomic study evidenced continuous WNV-L2 circulation in Italy throughout the year [29], while a reintroduction event was identified from Europe to Senegal, highlighting a potential threat [12].

Genomic characterization is even more important because it has been shown that West African lineages have higher virulence and replicative efficiency in vitro and in vivo compared to similar lineages circulating in the United States and Europe [6]. Genomic surveillance is thus essential as it allows a better understanding of the dissemination and dynamic of WNV strains.

In order to ensure the sustainability of this type of surveillance, we describe here the development and evaluation of a whole-genome amplicon-based sequencing approach for WNV-L1 and WNV-L2 by Illumina technology in different types of vertebrate and mosquito species from Senegal and Italy.

Three sets of primers were then designed and assessed with WNV-L1 and WNV-L2 strains. Set A and set B are specific to WNV-L1 and WNV-L2 strains, respectively, while the third one, a mixture of the two previous sets, is able to amplify both lineages.

Thus, the use of one set or another depends on the context. Indeed, in the case where the lineage is already well defined, it is appropriate to use the specific sets, whereas set A + B fits more in a context where no lineage characterization could be made before sequencing.

The evaluation in this study could only be carried out with the WNV-L1 and WNV-L2 strains. Because set A was designed from at least one representative of all the WNV lineages, it would be appropriate to undertake a similar evaluation with at least set A and set A + B on other lineages than WNV-L1 and WNV-L2. Moreover, the repetition of these experiments by other groups allows the observed results to be refined, particularly in terms of correlation with Ct values. Indeed, even if this work was carried out with rigor and with two teams in Senegal and Italy, external factors such as the sample quality after long-term storage or the sample type may have impacted the outputs of the results.

In any case, the approach presented in this manuscript could be a valuable tool for any WNV genomic investigation.

## Figures and Tables

**Table 1:** Sequences of the West Nile virus primers sets A and B.

WNV Primers Set A		WNV Primers Set B	
WNV_1_LEFT	GCCTGTGTGAGCTGACAAACTT	WNV-L2_1_LEFT	GCCTGTGTGAGCTGACAAACTT
WNV_1_RIGHT	TTCTTTTGTTTTGAGCTCCKCC	WNV-L2_1_RIGHT	TTCTTTTGTTTTGTGCTCCGGC
WNV_2_LEFT	ACAGCGATGAAACACCTTCTGA	WNV-L2_2_LEFT	ACAGCGATGAAGCATCTCTTGA
WNV_2_RIGHT	CGTGTCTTGGTGCATCTTCCAT	WNV-L2_2_RIGHT	GBCGDGTYTTDGTGCATCTYCC
KOUV_2_RIGHT	TTYCCTCTGATGCATCTTCCAT	WNV-L2_3_LEFT	GTSYTRGCTGCTGGAAATGAYC
WNV_3_LEFT	CCRGTACTGTCGGCTGGTAATG	WNV-L2_3_RIGHT	CMACCCATGTAGCTCCAGAYAC
WNV_3_RIGHT	CVAGAACCAAATCCACCCAWGT	WNV-L2_4_LEFT	ATNCTATTGCTCCTGGTRGCA
WNV_4_LEFT	ACAGCTTCAACTGCCTTGGAAT	WNV-L2_4_RIGHT	TCCADCCAGTTGCTTTGGTKGW
WNV_4_RIGHT	TGRTTCTTCCTATTGCCTTGGT	WNV-L2_5_LEFT	GTRGACAGRGGATGGGGAAAYG
WNV_5_LEFT	GGYTGCGGACTATTTGGMAAA	WNV-L2_5_RIGHT	GRTTCCTCCAHGYGGTGCTT
WNV_5_RIGHT	CTCCACACAGTACTTCCAGCAC	WNV-L2_6_LEFT	CCTTCCTGGTYCACCGAGARTG
WNV_6_LEFT	GGHACAAAGACGTTCTTGGTYC	WNV-L2_6_RIGHT	KGAVGAAATGGGCACYTTRCAR
WNV_6_RIGHT	GMACTTTGCAAGGTCCATCYGT	WNV-L2_7_LEFT	GCDTTYAAATTCGCYGGGACTC
WNV_7_LEFT	AYGCTTTCAAGTTTCTTGGGACT	WNV-L2_7_RIGHT	GTGTATRGCTTTCCCYACYGAG
KOUV_7_LEFT	AYGCTTTCAAGTTTCTTGGGCAT	WNV-L2_8_LEFT	ACTCAGAGGAGCTCAACGACTC
WNV_7_RIGHT	AASACTTGATGGACAGCCTTCC	WNV-L2_8_RIGHT	ATTYTTGCTAGGCCTTGTGGHG
WNV_8_LEFT	ARMGACTAGCCGCTCTAGGAGA	WNV-L2_9_LEFT	CGGTGYGGAAGTGGAGTGTTYA
WNV_8_RIGHT	TGDGCTTTCTGAATGATCTTGGCT	WNV-L2_9_RIGHT	TRYTRTTCCATGCTCGGTTSR
WNV_9_LEFT	AAYGATGTGGAGGCTTGGATGG	WNV-L2_10_LEFT	YGCDCCAGARCTAGCTAACAAYA
WNV_9_RIGHT	AWRCTATTCCAAGCGCGATTSK	WNV-L2_10_RIGHT	CCCTGGYCTCCTGTTRTGRTTGC
WNV_10_LEFT	TMTTTGCACCAGAACTCGCYAA	WNV-L2_11_LEFT	CACHYTGTGGGGTGATGGAGTT
WNV_10_RIGHT	CWGGTCTCCGATTGTGATTGCT	WNV-L2_11_RIGHT	CAGAAGGCCCAACTGAAAAGGA
WNV_11_LEFT	AYACCTTGTGGGGCGATGGART	WNV-L2_12_LEFT	GAYGAAAAGACCCTCGTGCA
WNV_11_RIGHT	GGCCCAACTGAAAAGGGTCAAT	WNV-L2_12_RIGHT	CCATTTGRAAGAAAGCAGCTGCR
WNV_12_LEFT	TGGARATCAGACCACAGAGRCA	WNV-L2_13_LEFT	CAGTCTTTCTGGTGGCTTCBTT
WNV_12_RIGHT	TTGRAAGAAAACAGCCGCCARC	WNV-L2_13_RIGHT	YCCAGCKGCAAGTATCATBGGA
WNV_13_LEFT	CCAGTGTTTATGGTGGCWTCGT	WNV-L2_14_LEFT	MRGTTGGAAGCYTCATCAARGARA
WNV_13_RIGHT	CHGCAAGGATCATGGGGTTGAA	WNV-L2_14_RIGHT	TGAAAATTTCCATCRTCATCCARCC
WNV_14_LEFT	GVAGCTTGATCAGGGAGAARAG	WNV-L2_15_LEFT	GGACRGCTGAYATYACYTGGGA
WNV_14_RIGHT	TCCBGGATCATTCATGARCTGG	WNV-L2_15_RIGHT	RCTCATGAGAGCRGCTCCCTTR
WNV_15_LEFT	CKGACATTTCCTGGGAAAGTGA	WNV-L2_16_LEFT	ATHATGACTCGAGGTCTGCTYG
WNV_15_RIGHT	TCATCAAAGCGGCTCCTTTWGT	WNV-L2_16_RIGHT	MRCCATTDGGCATGATGACKCC
WNV_16_LEFT	CTYGGCAGTTATCAAGCAGGAG	WNV-L2_17_LEFT	RGACTATCCCACYGGAACRTCA
WNV_16_RIGHT	WATBGCGCTTATGTATGAHCCR	WNV-L2_17_RIGHT	TGGCACATGACATCAACGATYT
WNV_17_LEFT	CMATAGTGGACAAAAACGGTGATGT	WNV-L2_18_LEFT	TKAGAGGACTTCCCATYCGGTA
WNV_17_RIGHT	DGTGAGGGTAGCATGACACATG	WNV-L2_18_RIGHT	TTBCCCATTTTCACACTTGGAACR
WNV_18_LEFT	ATGKCTGAAGCACTGAGRGGA	WNV-L2_19_LEFT	ACMGAGCCTGGAACACTGGVTA
WNV_18_RIGHT	CCCATCTTGACACTAGGCACAA	WNV-L2_19_RIGHT	CCTCCATAGCAATACTCATCACCA
WNV_19_LEFT	CGRGCTTGGAACTCTGGATAYG	WNV-L2_20_LEFT	TGATGGAAGAGTCATCCTGGGV
WNV_19_RIGHT	TACTCATCACCAACTTGCGAYG	WNV-L2_20_RIGHT	GTGTTGGTTCGAGGTCCATCAA
WNV_20_LEFT	GGAGAACCATCTGCAGTGACAG	WNV-L2_21_LEFT	CWGTCTGGCTCGCTTACAAAGT
WNV_20_RIGHT	CMACTTCGTTGTTGTCTTCTAAAATTG	WNV-L2_21_RIGHT	ARGCTATTGTCTGAAGGGCRTC
WNV_21_LEFT	CYTACAAGGTTGCAGCRGCT	WNV-L2_22_LEFT	TTKGACACGATGTATGTGGTKG
WNV_21_RIGHT	ACTCCCATGGTCATCACACWCA	WNV-L2_22_RIGHT	CAYTCTTGGTCTTGTCCAGCCA
WNV_22_LEFT	GAGGMAGAGCTCACAGAATGGC	WNV-L2_23_LEFT	YCAGCTYGCCGTGTTTTTGATY
WNV_22_RIGHT	CCTTGACCTCAATTCTTTGCCC	WNV-L2_23_RIGHT	CYGCAGTCACAGTCACAGTCAG
WNV_23_LEFT	TTGTGTCATGACCCTTGTSAGC	WNV-L2_24_LEFT	YTTTGTVGACGTTGGTGTGTCA
WNV_23_RIGHT	GGDACCATGTAGGCATAGTGGC	WNV-L2_24_RIGHT	TYGTTGCRTTCCACACTGARCT
WNV_24_LEFT	TTYGTCGATGTTGGAGTKTCA	WNV-L2_25_LEFT	ARRACTGTCAGAGAGGCYGGAA
WNV_24_RIGHT	GTYGTTGCGTTCCAMACWGAGC	WNV-L2_25_RIGHT	GCCTTTCCACTAACCACCGYAR
WNV_25_LEFT	RGACHGTVCGAGAAGCYGGAAT	WNV-L2_26_LEFT	RHGCCAGGAGAGAGGGAAAYRT
WNV_25_RIGHT	GRTCGAGGAAACBCCGTTCGAC	WNV-L2_26_RIGHT	CCARTCTTCCACCATCTCCARR
WNV_26_LEFT	RAGAAGGCAAYRTCACYGGAGG	WNV-L2_27_LEFT	CACACTGCTCTGTGACATTGGA
WNV_26_RIGHT	ARAATTCCCTTGGCCCTCGG	WNV-L2_27_RIGHT	AGAATTGAGGAGAGGCTTCCCY
WNV_27_LEFT	TCAAGTGCTGAGGTTGAAGAGC	WNV-L2_28_LEFT	AAGAAAACVTGGAAGGGACCYC
WNV_27_RIGHT	GCCTGAGTCGTTCAATCCTGTT	WNV-L2_28_RIGHT	TBGTGGTCTCATTGAGGACGTR
WNV_28_LEFT	TGTAAACTTGGGAAGTGGAACCA	WNV-L2_29_LEFT	CTCCTTTCGGHCAACAACGRGT
WNV_28_RIGHT	TTTWTCKCTGGCCAYAAAVGCC	WNV-L2_29_RIGHT	CYCCBAGCCACATGAACCADAT
WNV_29_LEFT	GTGGAYACGAAAGCTCCTGARC	WNV-L2_30_LEFT	ACYTGCATCTACAACATGATGGG
WNV_29_RIGHT	ATTGAGAAAACCSAGAGCTTCG	WNV-L2_30_RIGHT	GGBCTCATCACTTTCACGACYT
WNV_30_LEFT	MAARGCCAARGGMAGCAGAG	WNV-L2_31_LEFT	CDAAGGTBCTTGARCTGCTDGR
WNV_30_RIGHT	CCYCTCTGATCTTCTCTGGAGA	WNV-L2_31_RIGHT	GGACCTTTGACATGGCATTBAGR
WNV_31_LEFT	TGAGCTCACCTATCGWCACAAA	WNV-L2_32_LEFT	GGHGATGACTGYGTGGTDAA
WNV_31_RIGHT	CAYCCAGTTGACGGTTTCCACT	WNV-L2_32_RIGHT	GRACCCAGTTVACAGGCACA
WNV_32_LEFT	TGGTRAAGCCCCTGGAYGAY	WNV-L2_33_LEFT	GCAGATGTGGCTGYTGCTTTAT
WNV_32_RIGHT	TCTCCTCCTGCATGGATSGA	WNV-L2_33_RIGHT	YRTCTTCATACCTCCTCARDGA
WNV_33_LEFT	AGWAGAGACCTGMGGYTCAT	WNV-L2_34_LEFT	GCGCHACTTGGGCTGAAAAYAT
WNV_33_RIGHT	TCTACAAAACTGTGTCCTCAACCA	WNV-L2_34_RIGHT	MYCTTCCGAGACGGTTCTGA
WNV_34_LEFT	AGTCAGWKCAATCATCGGRGAWG	WNV-L2_35_LEFT	GGAAGTTGAGTAGACGGTGCTG
WNV_34_RIGHT	CACTATCGCAGACTGCACTCTC	WNV-L2_35_RIGHT	TCCCAGGTGTCAATATGCTGTT
WNV_35_LEFT	CAGGAGGACTGGGTGAACAAAG		
WNV_35_RIGHT	TGGTTGTGCAGAGCAGAAGATC		

**Table 2:** Inclusivity test of the West Nile virus set A primers.

RT-PCR Ct Value		Total Number of Trimmate Reads	Number of WNV Reads	% HCoverage	VCoverage	Consensus Sequence Length
Viral strain WNV L1 Italy	14	2369.723	649.022	99%	5802.67	10.969
	14	1922.532	581.215	99%	5346.85	10.966
	13	2715.830	754.081	99%	6244.36	10.966
	No. of replicates with coverage ≥ 95%			3/3 (100%)		
Viral strain WNV L1 Senegal	25	623.535	238.259	99%	7997	10.961
	28	5034.151	1035.983	99%	6862.12	10.965
	16	5034.151	547.775	99%	5183.83	10.966
	19	810.906	327.395	100%	3971.4	11.018
	17	924.142	363.236	99%	4412.7	10.963
	17	899.818	342.133	99%	3851.34	10.966
	17	819.552	358.631	99%	4306.94	10.961
	No. of replicates with coverage ≥ 95%			7/7 (100%)		
Viral strain WNV L2 Italy	18	2607.185	546.249	100%	3607.99	10.926
	18.71	2466.682	511.381	100%	3488.1	10.926
	18.13	2061.961	465.844	100%	3445.55	10.926
	No. of replicates with coverage ≥ 95%			3/3 (100%)		
Viral strain WNV L2 Senegal	14	4861.644	706.114	100%	3519.51	10.914
	14	4087.737	835.098	100%	5593.58	10.914
	14	4750.250	885.595	100%	5609.34	10.914
	No. of replicates with coverage ≥ 95%			3/3 (100%)		

**Table 3:** Sensitivity test of the West Nile virus set A primers.

Viral Strain	Quantity Value (cp/μL)	Quantity Mean Value (Ct)	Total Number of Trimmate Reads	Number of WNV Reads	% HCoverage	VCoverage	Consensus Sequence Length
WNV L1 (reference used for the mapping on Genpat: WNV L1 FJ483548	10^6^	18.68	1457.278	503.152	99%	5043.56	10.959
	10^6^		1371.693	476.605	99%	4997.04	10.964
	10^6^		938.406	375.653	99%	4372.01	10.959
	No. of replicates with coverage ≥ 95%				3/3 (100%)		
	10^5^	23.45	1342.407	426.914	99%	4587.33	10.960
	10^5^		1174.155	397.215	99%	4435.79	10.959
	10^5^		825.135	315.219	99%	3776.67	10.956
	No. of replicates with coverage ≥ 95%				3/3 (100%)		
	10^4^	27	906.690	292.718	96%	3456.26	10.964
	10^4^		984.952	297.351	97%	3414.38	10.959
	10^4^		1247.127	327.095	96%	3570.22	10.959
	No. of replicates with coverage ≥ 95%				3/3 (100%)		
	10^3^	30	1096.401	249.798	94%	2735.41	10.962
	10^3^		506.380	153.284	93%	1853.42	10.955
	10^3^		569.039	170.272	91%	2099.54	10.955
	No. of replicates with coverage ≥ 95%				0/3 (0%)		
	10^2^	33	57.229	20.115	82%	295.76	10.366
	10^2^		53.435	18.515	81%	281.027	10.958
	10^2^		41426	15.149	80%	227.617	10.951
	No. of replicates with coverage ≥ 95%				0/3 (0%)		
WNV L2 (reference used for the mapping: WNV L2 MN652880)	10^6^	18.68	1262.167	12.484	35%	391.547	10.028
	10^6^		1315.568	11.576	33%	383.276	10.928
	10^6^		1103.838	16.652	26%	706.146	10.928
	No. of replicates with coverage ≥ 95%				0/3 (0%)		
	10^5^	22.87	1210.495	11.251	36%	347.197	10.928
	10^5^		823.025	10.243	33%	345.101	10.928
	10^5^		1478.160	12.253	34%	394.675	10.928
	No. of replicates with coverage ≥ 95%				0/3 (0%)		
	10^4^	26.41	1228.945	9.715	32%	337.862	10.928
	10^4^		1090.849	9.547	32%	327.57	10.928
	10^4^		947.964	4.405	30%	159.122	10.928
	No. of replicates with coverage ≥ 95%				0/3 (0%)		
	10^3^	30	442.063	2.796	26%	121.744	10.928
	10^3^		577.277	3.500	27%	143.439	10.926
	10^3^		369.041	1.615	22%	79.118	10.245
	No. of replicates with coverage ≥ 95%				0/3 (0%)		
	10^2^	33.14	35.426	1.362	17%	789.157	10.924
	10^2^		68.307	627	17%	360.495	10.926
	10^2^		63.643	731	18%	571.297	10.926
	No. of replicates with coverage ≥ 95%				0/3 (0%)		

**Table 4:** Test of the West Nile virus set A primers with WNV-L1 homogenates. (* Samples with multiple viral species/WNV lineage).

Viral Homogenate WNV L1—Sample Number	Host	RT-PCR Ct Value	Co-InfectionCt Value	# Total Trimmate Reads	# WNV L1 Reads	% HCoverage	VCoverage	Consensus Sequence Length
1	*Accipiter gentilis*	15	-	2,037,215	591,954	100%	6362.04	11,027
2	*Pica pica*	16	-	14,648,025	1,333,187	100%	6381.67	11,016
3	*Corvus cornix*	16	-	6,447,209	657,948	99%	5135.45	10,966
4	*Pica pica*	18	-	2,923,237	387,986	99%	4264.56	10,966
5	*Phalacrocorax carbo*	19	-	11,441,518	1,107,291	99%	5888.49	10,961
6	*Corvus cornix*	19	-	2,347,380	351,292	99%	4177.69	10,963
7	*Culex pipiens*	20	-	3,495,685	744,562	99%	5560.68	10,968
8	*Culex pipiens*	22	-	7,571,826	597,499	98%	3973.2	10,967
9	*Corvus cornix*	22	-	5,382,363	629,364	99%	4806.11	10,960
10	*Passer domesticus*	22	-	3,942,375	304,215	97%	3084.4	10,962
11	*Culex pipiens*	23	-	4,560,711	283,771	93%	2899.47	10,960
12	*Corvus Cornix*	24	-	3,052,530	342,271	93%	3508.25	10,966
13 *	*Culex pipiens*	25	L2 Ct 28	3,576,787	403,166	94%	3761.48	10,966
14 *	*Culex pipiens*	25	L2 Ct 28	1,557,373	263,149	90%	2471.3	10,954
15	*Larus michahellis*	25	-	1,813,567	129,834	88%	1805.95	10,952
16	*Streptopelia decaocto*	26	-	1,439,897	203,172	91%	2592.09	10,961
17	*Pica pica*	26	-	7,677,172	251,982	81%	2920.03	10,963
18	*Parus major*	26	-	755,951	38,780	69%	747.61	9389
19 *	*Culex pipiens*	27	L2 Ct 32	3,371,541	369,610	96%	3287.31	10,956
20	*Turdus merula*	27	-	2,710,572	66,229	82%	1039.08	10,954
21	*Culex pipiens*	28	-	6,865,408	269,727	94%	2618.84	10,962
22 *	*Culex pipiens*	28	L2 Ct 31	3,378,716	190,102	87%	2201.29	10,966
23	*Streptopelia decaocto*	28	-	1,842,010	22,006	68%	430.494	10,946
24	*Equus caballus*	28	-	1,805,957	159,703	92%	2101.31	10,960
25	*Athene noctua*	29	-	3,394,989	15,767	34%	404	10,802
26	*Columba palumbus*	31	-	835,362	9948	57%	211.706	10,960
27 *	*Culex pipiens*	33	L2 Ct 25	4,662,340	220,508	91%	2381.21	10,963
28 * (Alphavirus, Mesonivirus)	*Culex neavei*	28.5	-	391,494	1,135,180	92.42%	2514.3	10,194
29 * (Barkedji, Mesonivirus)	*Culex poicilipes*	35.59	-	64,716	616,500	82.35%	1789.36	9083
30 * (Barkedji)	*Culex neavei*	29.52	-	302,175	1,394,466	89%	2001.3	9819
31 * (Alphavirus, Barkedji, Usutu)	*Culex neavei*	25.03	-	180,791	484,221	73.89%	822.02	8150

**Table 5:** Test of the West Nile virus set A primers with WNV-L2 homogenates. (* Samples with multiple viral species/WNV lineage).

Viral Homogenate WNV L2—Sample Number	Host	RT-PCR Ct Value	Co-Infection Ct Value	# Total Trimmate Reads	# WNV Reads	% HCoverage	VCoverage	Consensus Sequence Length
1	*Accipiter gentilis*	16	-	3336.278	450.617	100%	3304.6	10.926
2	*Accipiter gentilis*	16	-	1773.333	393.125	99%	2982.38	10.926
3	*Accipiter gentilis*	19	-	2753.185	225.096	72%	1533.63	10.926
4	*Garrulus glandarius*	20	-	3540.566	304.094	96%	1588.44	10.923
5	*Culex pipiens*	22	-	1092.527	517.44	58%	779.912	10.921
6	*Culex pipiens*	23	-	568.562	54.658	59%	905.155	10.834
7	*Corvus cornix*	23	-	1383.831	85.880	62%	1168.95	10.922
8	*Passer italiae*	24	-	476.120	45.644	47%	1089.79	10.923
9	*Columba palumbus*	26	-	1313.900	76.773	64%	908.822	10.923
10	*Columba palumbus*	27	-	5.377	7.319	25%	257.256	10.868
11	*Columba palumbus*	28	-	545.944	5073	23%	281.009	8.425
12	*Turdus merula*	31	-	514.655	72	4%	26.336	375
13	*Pica pica*	31	-	330.374	280	5%	685.197	4.128
14	*Phasianus colchicus*	32	-	243.656	242	5%	543.463	7.510
15	*Pica pica*	33	-	341.153	613	9%	760.203	8.413
16	*Pica pica*	33	-	376.185	128	4%	307.715	3.779
17	*Egretta garzetta*	34	-	616.781	395	6%	715.396	7.923
18	*Culex pipiens*	29	-	1537.448	24.517	33%	676.501	9.393
19	*Culex pipiens*	28	-	588.313	32.437	49%	749.224	10.924
20	*Culex pipiens*	27	-	606.886	28.181	52%	494.551	10.923
21	*Culex pipiens*	25	-	884.756	43.657	52%	864.517	10.891
22	*Culex pipiens*	30	-	259.173	2.572	3%	989.276	381
23	*Culex pipiens*	28	-	436.461	14.473	29%	630.097	10.923
24	*Culex pipiens*	24	-	2172.952	45.568	45%	716.974	10.923
25 *	*Culex pipiens*	31	L1 Ct 28	3378.716	9.132	27%	447.986	10.928
26	*Culex pipiens*	25	-	339.617	33.681	42%	827.229	10.790
27	*Culex pipiens*	21	-	1223.422	35.451	23%	1197.74	10.921
28	*Culex pipiens*	23	-	1832.341	38.134	15%	1569.34	10.555
29	*Culex pipiens*	25	-	686.602	25.509	18%	1178.55	10.609
30	*Culex pipiens*	27	-	502.162	8.253	3%	3113.1	388
31	*Corvus cornix*	28	-	1357.321	7.159	31%	312.307	10.475
32	*Pica pica*	20	-	6417.489	198.613	94%	1616.11	10.927
33 *	*Culex pipiens*	25	L1 Ct 33	4662.340	33.608	60%	534.955	10.926
34 *	*Culex pipiens*	24	USUV Ct 27	5133.183	77.022	61%	888.532	10.925
35	*Culex pipiens*	24	-	153.601	10	47%	275.337	556
36	*Corvus cornix*	29	-	877.218	0	0%	0	0
37	*Culex pipiens*	23	-	199.550	99.490	88%	1126.14	10.890
38	*Culex pipiens*	24	-	323.115	5.048	23%	308.136	4.395
39 *	*Culex pipiens*	27	L1 Ct 25	3576787	9.461	51%	239.312	10.928
40	*Culex pipiens*	22	-	78.378	42.637	63%	824.461	10.922
41	*Larus marinus*	23	-	546.215	142.178	81%	1460.97	10.923
42 *	*Culex pipiens*	27	L1 Ct 32	3371.541	5.603	47%	154.32	10.928
43	*Culex pipiens*	29	-	153.413	4.367	31%	187.09	9.364
44	*Culex pipiens*	28	-	66.202	7.786	36%	290.742	10.018
45 *	*Culex pipiens*	28	L1 Ct 25	1557.373	8.945	48%	243.941	10.926
46	*Culex pipiens*	28	-	118.396	2.420	15%	219.733	7.809
47 *	*Culex pipiens*	27	USUV Ct 27	1516.324	230	44%	985.194	7.469
48	*Corvus cornix*	28	-	470.113	6.194	40%	207.827	10.923
49 *	*Culex pipiens*	23	USUV Ct 21	4272.994	248	29%	941.936	10.806
50	*Culex pipiens*	15	-	327.238	88.896	61%	1398.22	10.922
51	*Ochlerotatus caspius*	25	-	361.194	16.436	32%	684.576	10.844
52	*Culex pipiens*	24	-	401.408	25.091	42%	804.597	9.592
53	*Pica pica*	23	-	899.597	2.771	24%	150.215	8.402
54 *	*Culex pipiens*	29	USUV Ct 26	117.768	18.204	54%	451.546	10.907

**Table 6:** Inclusivity test of the West Nile virus set B primers.

RT-PCR Ct Value		# Total Trimmate Reads	# WNV Reads	% HCoverage	VCoverage	Consensus Sequence Length
Viral strain WNV L2 Italy	15	1218.086	284.232	100%	3820.77	10.926
	15	1792.478	402.082	100%	5234.36	10.926
	15	1440.061	338.706	100%	4543.81	10.926
	17	1711.005	328.182	100%	4374.41	10.926
	18	941.641	224.716	100%	3023.77	10.926
	N of replicates with Coverage ≥ 95%			5/5 (100%)		

**Table 7:** Sensitivity test of the West Nile virus set B primers.

Viral Strain	Quantity Value (cp/5 μL)	Quantity Mean Value (Ct)	# Total Trimmate Reads	# WNV Reads	% HCoverage	VCoverage	Consensus Sequence Length
WNV L2 (reference used for the mapping: WNV L2 MN652880)	10^6^	19	1808.185	381.930	100%	5038.73	10.913
	10^6^		4928.502	673.900	100%	6845.55	10.926
	10^6^		3099.180	511.446	100%	6107.38	10.913
	No. of replicates with coverage ≥ 95%				3/3 (100%)		
	10^5^	22	2665.260	409.989	100%	5157.74	10.914
	10^5^		1049.020	237.471	100%	3194.36	10.926
	10^5^		2820.387	429.662	100%	5335.99	10.912
	No. of replicates with coverage ≥ 95%				3/3 (100%)		
	10^4^	26	1651.945	261.024	100%	3450.12	10.913
	10^4^		2483.786	337.982	100%	4234.91	10.914
	10^4^		2681.807	356.233	100%	4334.71	10.926
	No. of replicates with coverage ≥ 95%				3/3 (100%)		
	10^3^	30	1570.036	236.060	100%	3029.53	10.894
	10^3^		1153.288	196.257	100%	2614.91	10.904
	10^3^		782.424	285.136	99%	3070.47	10.912
	No. of replicates with coverage ≥ 95%				3/3 (100%)		
	10^2^	33	1764.307	159.591	95%	2189.31	10.597
	10^2^		2082.360	177.212	93%	2389.54	10.800
	10^2^		NA	NA	NA	NA	NA
	No. of replicates with coverage ≥ 95%				1/2 (50%)		

**Table 8:** Test of the West Nile virus set B primers with WNV-L2 homogenates.

Viral Homogenate WNV L2—Sample Number	RT-PCR Ct Value	Host	Total Number of Trimmate Reads	Number of WNV Reads	% HCoverage	VCoverage	Consensus Sequence Length
1	17	*Pica pica*	1134.961	295.649	100%	3977.58	10.892
2	27	*Corvus cornix*	1448.962	284.947	100%	3818.15	10.912
3	21	*Culex pipiens*	1733.553	385.088	100%	5042.35	10.913
4	23	*Culex pipiens*	1528.111	339.207	100%	4525.72	10.912
5	21	*Athene noctua*	1801.390	376.213	100%	4946.53	10.878
6	22	*Culex pipiens*	1407.784	312.376	99%	4201.89	10.879
7	19	*Passer domesticus*	1515.878	205.605	100%	2770.45	10.914
8	30	*Corvus cornix*	1470.367	209.316	98%	2799.57	10.872
9	30	*Pica pica*	2150.396	205.466	99%	2662.61	10.868
10	27	*Sylvia atricapilla*	3649.208	281.102	99%	3426.07	10.880
11	25	*Culex pipiens*	2094.249	349.710	100%	4076.35	10.878
12	29	*Anopheles maculipennis*	3435.959	563176	100%	5709.14	10.912
13	25	*Culex pipiens*	3120.601	281.102	100%	4601.81	10.912
14	27	*Culex pipiens*	2193.040	259.483	97%	3231.74	10.904
15	35	*Equus ferus caballus*	1623.442	133.088	93%	1814.74	10.936

**Table 9:** Test of the West Nile virus set A + B primers with WNV-L1 and WNV-L2 homogenates.

Viral Homogenate WNV L1	RT-PCR Ct Value	Host	Used Primers	Total Number of Trimmate Reads	Number of WNV Reads	% HCoverage	VCoverage	Consensus Sequence Length
1	L1 19	*Corvus cornix*	Set A	2347.380	351.292	99%	4177.69	10.963
			Set A + B	1118.615	287.672	99%	3987.12	10.963
2	L1 25	*Larus michahellis*	Set A	1813.567	129.834	88%	1805.95	10.952
			Set A + B	651.723	91.642	93%	1305.83	10.960
3	L1 18	*Pica pica*	Set A	2923.237	387.986	99%	4264.56	10.966
			Set A + B	4965.722	512.725	99%	4562.28	10.966
4	L1 28	*Equus ferus caballus*	Set A	1805.957	159.703	92%	2101.31	10.960
			Set A + B	2319.560	165.376	92%	2200.39	10.960
**Viral Homogenate WNV L2**	**RT-PCR Ct Value**	**Host**	**Used Primers**	**Total Number of Trimmate Reads**	**Number of WNV Reads**	**% HCoverage**	**VCoverage**	**Consensus Sequence Length**
1	L2 17	*Pica pica*	Set B	2059.659	351.670	100%	4232.78	10.892
			Set A + B	1134.961	295.649	100%	3977.58	10.892
2	L2 27	*Corvus cornix*	Set B	1793.046	187.826	100%	2156.81	10.926
			Set A + B	1448.962	284.947	100%	3818.15	10.912
3	L2 21	*Culex pipiens*	Set B	2219.785	312.851	100%	3515.05	10.926
			Set A + B	1733.553	385.088	100%	5042.35	10.913
4	L2 23	*Culex pipiens*	Set B	2045.957	275.354	100%	2943.77	10.926
			Set A + B	1528.111	339.207	100%	4525.72	10.912
5	L2 21	*Athene noctua*	Set B	3413.467	359.916	100%	4106.39	10.892
			Set A + B	1801.390	376.213	100%	4946.53	10.878
6	L2 22	*Culex pipiens*	Set B	3109.597	239.795	95%	2761.23	10.892
			Set A + B	1407.784	312.376	99%	4201.89	10.879
7	L2 19	*Passer domesticus*	Set B	1621.442	123.813	89%	1836.85	10.924
			Set A + B	1515.878	205.605	100%	2770.45	10.914

## Data Availability

The data presented in this study are available in the results section of the article as well as in the supplementary materials. No new data were created or analyzed in this study.

## Supplementary Materials

The following supporting information can be downloaded at: https://www.mdpi.com/article/10.3390/v15061261/s1, Table S1: WNV Sequences aligned for set A primers design, Table S2: WNV-L2 Sequences aligned for set B primers design.
